# *trans*-Bis(thio­cyanato-κ*N*)tetra­kis­(3,4,5-trimethyl-1*H*-pyrazole-κ*N* ^2^)nickel(II)–3,4,5-trimethyl-1*H*-pyrazole (1/1)

**DOI:** 10.1107/S1600536811041419

**Published:** 2011-10-29

**Authors:** Moayad Hossaini Sadr, James T. Engle, Christopher J. Ziegler, Behzad Soltani, Zahra Mousavi

**Affiliations:** a Department of Chemistry, Azarbaijan University of Tarbiat Moallem, Tabriz, Iran; b Department of Chemistry, University of Akron, Akron, Ohio 44325, USA

## Abstract

In the title compound, [Ni(NCS)_2_(C_6_H_10_N_2_)_4_]·C_6_H_10_N_2_, the asymmetric unit comprises a Ni^II^ complex and a co-crystallised mol­ecule of 3,4,5-trimethyl-1*H*-pyrazole (PzMe_3_). The Ni^II^ atom is coordinated by four PzMe_3_ mol­ecules and two thio­cyanate anions to define a *trans* N_4_S_2_ distorted octa­hedral geometry. A number of intra­molecular N—H⋯N, N—H⋯S and C—H⋯N inter­actions contribute to the stability of the complex. The crystal structure is stabilized by inter­molecular N—H⋯S inter­actions, which link neighbouring mol­ecules into chains along the *a* axis.

## Related literature

For some background to imidazole in coordination chemistry, see: Hossaini Sadr *et al.* (2004 ▶, 2006 ▶, 2008 ▶); Wriedt *et al.* (2010 ▶).
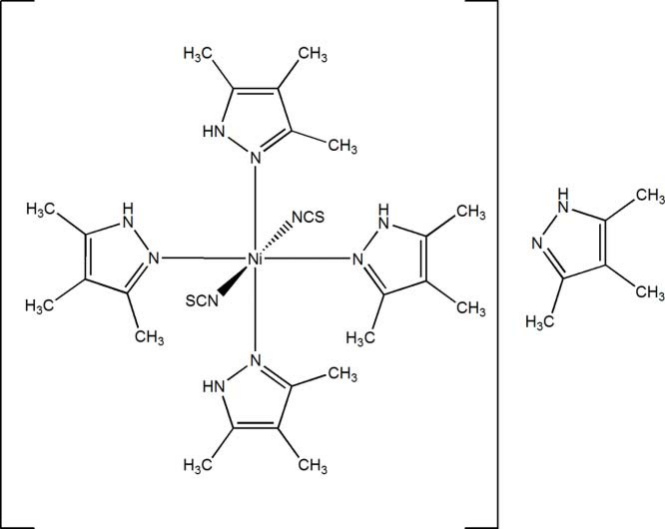


## Experimental

### 

#### Crystal data

[Ni(NCS)_2_(C_6_H_10_N_2_)_4_]·C_6_H_10_N_2_
*M* *_r_* = 725.67
Triclinic, 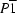

*a* = 8.640 (8) Å
*b* = 12.561 (11) Å
*c* = 19.30 (2) Å
α = 101.815 (15)°
β = 98.817 (16)°
γ = 107.895 (11)°
*V* = 1898 (3) Å^3^
*Z* = 2
Mo *K*α radiation
μ = 0.66 mm^−1^
*T* = 100 K
0.20 × 0.20 × 0.03 mm

#### Data collection

Bruker APEXII CCD diffractometer
Absorption correction: multi-scan (*SADABS*; Sheldrick, 1996 ▶) *T*_min_ = 0.879, *T*_max_ = 0.981
13255 measured reflections
6625 independent reflections
4497 reflections with *I* > 2σ(*I*)
*R*_int_ = 0.072

#### Refinement

*R*[*F* ^2^ > 2σ(*F* ^2^)] = 0.073
*wR*(*F* ^2^) = 0.240
*S* = 1.02
6625 reflections
439 parameters
H-atom parameters constrained
Δρ_max_ = 0.78 e Å^−3^
Δρ_min_ = −1.31 e Å^−3^

### 

Data collection: *APEX2* (Bruker, 2004 ▶); cell refinement: *SAINT* (Bruker, 2004 ▶); data reduction: *SAINT*; program(s) used to solve structure: *SHELXTL* (Sheldrick, 2008 ▶); program(s) used to refine structure: *SHELXTL*; molecular graphics: *SHELXTL*; software used to prepare material for publication: *SHELXTL* and *PLATON* (Spek, 2009 ▶).

## Supplementary Material

Crystal structure: contains datablock(s) global, I. DOI: 10.1107/S1600536811041419/tk2796sup1.cif

Structure factors: contains datablock(s) I. DOI: 10.1107/S1600536811041419/tk2796Isup2.hkl

Additional supplementary materials:  crystallographic information; 3D view; checkCIF report

## Figures and Tables

**Table 1:** Selected bond lengths (Å)

Ni1—N1	2.071 (5)
Ni1—N2	2.065 (5)
Ni1—N3	2.128 (5)
Ni1—N6	2.111 (5)
Ni1—N8	2.120 (5)
Ni1—N10	2.108 (5)

**Table 2:** Hydrogen-bond geometry (Å, °)

*D*—H⋯*A*	*D*—H	H⋯*A*	*D*⋯*A*	*D*—H⋯*A*
N5—H5⋯N11	0.86	2.15	2.970 (8)	159
N7—H7⋯S2^i^	0.86	2.66	3.441 (6)	152
N9—H9⋯S1^ii^	0.86	2.59	3.348 (6)	148
N12—H12⋯S1	0.86	2.49	3.292 (7)	156
C3—H3*A*⋯N2	0.98	2.57	3.338 (9)	135
C14—H14*A*⋯N1	0.98	2.50	3.324 (9)	141
C20—H20*A*⋯N1	0.98	2.49	3.371 (8)	150
C21—H21*A*⋯N2	0.98	2.48	3.326 (8)	145

Symmetry codes: (i) 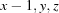; (ii) 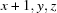.

**Fig. 1.**
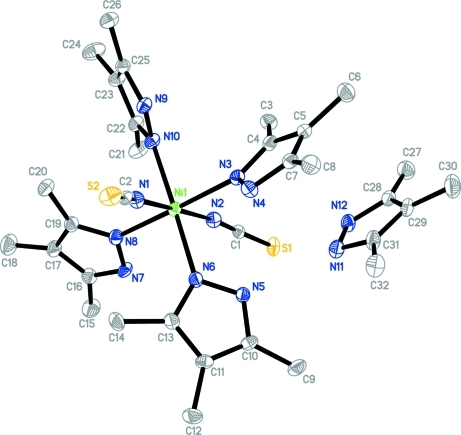
Molecular structure of the asymmetric unit in the title compound showing 40% probability displacement ellipsoids and the atomic numbering. H atoms have been removed for reasons of clarity.

**Fig. 2.**
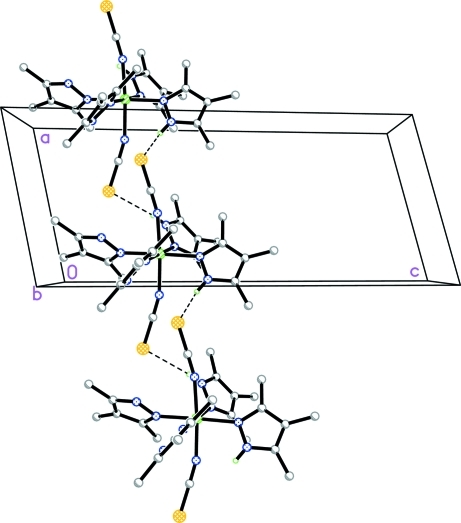
A partial packing diagram of the title compound viewed down the b axis showing an extended chain along the a axis through N—H···S interactions (dashed lines). Only the H atoms involved the H-bonding are shown.

## supplementary crystallographic information

### Comment

Complexes of pyrazole-based ligands are a frequent subject of chemical investigations and are used to better understand the relationship between structure and activity in the active sites of metalloproteins (Wriedt *et al.*, 2010). Currently, there is interest in designing various pyrazole-derived ligands with specific structural properties to fulfill the specific stereochemical requirements of a particular metal-binding site. In our systematic studies on transition metal complexes with pyrazole derivatives (Hossaini Sadr *et al.*, 2004; Hossaini Sadr *et al.*, 2008; Hossaini Sadr *et al.*, 2006), the title compound was prepared and its X-ray crystal structure was determined.

The asymmetric unit of the title complex, Fig. 1 and Table 1, comprises one molecule of the complex and a co-crystallized pyrazole ligand. The geometry around Ni is that of distorted octahedron and is coordinated by four 3,4,5-trimethyl-3*H*-pyrazole molecules and two thiocyanate anions. A number of intramolecular N—H···N, N—H···S and C—H···N interactions contribute to the stability of the complex. The crystal structure is stabilized by intermolecular N—H···S interactions which link neighbouring molecules into chains along the *a* axis (Fig. 2 and Table 2).

### Experimental

To a mixture of NiCl_2_.6H_2_O (0.1 g, 1 mmol) and Pz(Me)_3_ (0.185 g, 4 mmol) in acetone (30 ml), KSCN (0.08 g, 2 mmol) was added and the mixture was stirred for 12 h. The resultant solution was then filtered. The filtered solution was then stored for three days at 269 K after which blue plates formed.

### Refinement

All C-bound H atoms were positioned geometrically with C—H = 0.98 Å and included in a riding model approximation with *U*_iso_ (H) = 1.5 *U*_eq_(C). The N-bound H atoms were located from the difference Fourier map but were fixed with N—H = 0.86 Å, and refined with *U*_iso_(H) = 1.2*U*_eq_(N).

### Crystal data

**Table:**

[Ni(NCS)_2_(C_6_H_10_N_2_)_4_]·C_6_H_10_N_2_	*Z* = 2
*M**_r_* = 725.67	*F*(000) = 772
Triclinic, *P*1	*D*_x_ = 1.270 Mg m^−^^3^
Hall symbol: -P 1	Mo *K*α radiation, λ = 0.71073 Å
*a* = 8.640 (8) Å	Cell parameters from 2348 reflections
*b* = 12.561 (11) Å	θ = 2.2–23.6°
*c* = 19.30 (2) Å	µ = 0.66 mm^−^^1^
α = 101.815 (15)°	*T* = 100 K
β = 98.817 (16)°	Plate, blue
γ = 107.895 (11)°	0.20 × 0.20 × 0.03 mm
*V* = 1898 (3) Å^3^	

### Data collection

**Table:**

Bruker APEXII CCD diffractometer	6625 independent reflections
Radiation source: fine-focus sealed tube	4497 reflections with *I* > 2σ(*I*)
graphite	*R*_int_ = 0.072
φ and ω scans	θ_max_ = 25.0°, θ_min_ = 1.1°
Absorption correction: multi-scan (*SADABS*; Sheldrick, 1996)	*h* = −10→10
*T*_min_ = 0.879, *T*_max_ = 0.981	*k* = −14→14
13255 measured reflections	*l* = −22→22

### Refinement

**Table:**

Refinement on *F*^2^	Primary atom site location: structure-invariant direct methods
Least-squares matrix: full	Secondary atom site location: difference Fourier map
*R*[*F*^2^ > 2σ(*F*^2^)] = 0.073	Hydrogen site location: inferred from neighbouring sites
*wR*(*F*^2^) = 0.240	H-atom parameters constrained
*S* = 1.02	*w* = 1/[σ^2^(*F*_o_^2^) + (0.1309*P*)^2^] where *P* = (*F*_o_^2^ + 2*F*_c_^2^)/3
6625 reflections	(Δ/σ)_max_ < 0.001
439 parameters	Δρ_max_ = 0.78 e Å^−^^3^
0 restraints	Δρ_min_ = −1.31 e Å^−^^3^

### Special details

**Table:**

Experimental. Estimated minimum and maximum transmission: 0.5159 0.7457
Geometry. All e.s.d.'s (except the e.s.d. in the dihedral angle between two l.s. planes) are estimated using the full covariance matrix. The cell e.s.d.'s are taken into account individually in the estimation of e.s.d.'s in distances, angles and torsion angles; correlations between e.s.d.'s in cell parameters are only used when they are defined by crystal symmetry. An approximate (isotropic) treatment of cell e.s.d.'s is used for estimating e.s.d.'s involving l.s. planes.
Refinement. Refinement of *F*^2^ against ALL reflections. The weighted *R*-factor *wR* and goodness of fit *S* are based on *F*^2^, conventional *R*-factors *R* are based on *F*, with *F* set to zero for negative *F*^2^. The threshold expression of *F*^2^ > σ(*F*^2^) is used only for calculating *R*-factors(gt) *etc*. and is not relevant to the choice of reflections for refinement. *R*-factors based on *F*^2^ are statistically about twice as large as those based on *F*, and *R*- factors based on ALL data will be even larger.

### Fractional atomic coordinates and isotropic or equivalent isotropic displacement parameters (Å^2^)

**Table:**

	*x*	*y*	*z*	*U*_iso_*/*U*_eq_	
Ni1	0.82210 (8)	0.80401 (6)	0.72446 (4)	0.0256 (2)	
S1	0.23581 (18)	0.61334 (13)	0.71314 (9)	0.0378 (4)	
S2	1.41564 (18)	0.96437 (13)	0.82925 (8)	0.0377 (4)	
N1	1.0794 (6)	0.8855 (4)	0.7525 (2)	0.0309 (11)	
N2	0.5670 (6)	0.7197 (4)	0.7044 (2)	0.0314 (11)	
N3	0.8625 (6)	0.6637 (4)	0.7608 (2)	0.0270 (10)	
N4	0.9828 (6)	0.6877 (4)	0.8216 (2)	0.0305 (11)	
H4	1.0376	0.7567	0.8491	0.037*	
N5	0.7197 (6)	0.8244 (4)	0.8696 (2)	0.0285 (10)	
H5	0.6683	0.7505	0.8556	0.034*	
N6	0.8146 (6)	0.8857 (4)	0.8304 (2)	0.0310 (11)	
N7	0.6340 (6)	0.9627 (4)	0.6944 (2)	0.0314 (11)	
H7	0.5738	0.9364	0.7229	0.038*	
N8	0.7747 (6)	0.9386 (4)	0.6841 (2)	0.0305 (11)	
N9	0.9785 (5)	0.7043 (4)	0.6119 (2)	0.0282 (10)	
H9	1.0544	0.7099	0.6483	0.034*	
N10	0.8375 (6)	0.7269 (4)	0.6191 (2)	0.0302 (11)	
C1	0.4274 (7)	0.6768 (4)	0.7076 (3)	0.0251 (12)	
C2	1.2198 (7)	0.9184 (4)	0.7837 (3)	0.0270 (12)	
C3	0.6728 (7)	0.4835 (5)	0.6679 (3)	0.0352 (14)	
H3A	0.6323	0.5387	0.6484	0.053*	
H3B	0.5802	0.4272	0.6793	0.053*	
H3C	0.7174	0.4427	0.6315	0.053*	
C4	0.8071 (7)	0.5473 (5)	0.7351 (3)	0.0291 (12)	
C5	0.8928 (7)	0.4983 (5)	0.7804 (3)	0.0299 (13)	
C6	0.8723 (8)	0.3719 (5)	0.7691 (4)	0.0442 (16)	
H6A	0.9432	0.3625	0.8105	0.066*	
H6B	0.9053	0.3446	0.7243	0.066*	
H6C	0.7551	0.3263	0.7651	0.066*	
C7	1.0077 (7)	0.5912 (5)	0.8347 (3)	0.0310 (13)	
C8	1.1416 (8)	0.5985 (5)	0.8954 (3)	0.0411 (15)	
H8A	1.2510	0.6359	0.8860	0.062*	
H8B	1.1311	0.5202	0.8993	0.062*	
H8C	1.1314	0.6443	0.9410	0.062*	
C9	0.6137 (8)	0.8441 (5)	0.9821 (3)	0.0413 (15)	
H9A	0.5707	0.7590	0.9656	0.062*	
H9B	0.5199	0.8724	0.9818	0.062*	
H9C	0.6838	0.8694	1.0316	0.062*	
C10	0.7156 (7)	0.8919 (5)	0.9321 (3)	0.0294 (12)	
C11	0.8115 (7)	1.0035 (5)	0.9359 (3)	0.0306 (13)	
C12	0.8445 (8)	1.1103 (5)	0.9962 (3)	0.0405 (15)	
H12A	0.7680	1.0920	1.0282	0.061*	
H12B	0.8268	1.1717	0.9755	0.061*	
H12C	0.9603	1.1367	1.0244	0.061*	
C13	0.8712 (7)	0.9970 (5)	0.8722 (3)	0.0315 (13)	
C14	0.9780 (8)	1.0950 (5)	0.8486 (3)	0.0424 (16)	
H14A	1.0240	1.0640	0.8090	0.064*	
H14B	1.0697	1.1467	0.8898	0.064*	
H14C	0.9103	1.1385	0.8316	0.064*	
C15	0.4515 (9)	1.0713 (6)	0.6552 (4)	0.0557 (19)	
H15A	0.3498	1.0073	0.6265	0.084*	
H15B	0.4671	1.1376	0.6343	0.084*	
H15C	0.4411	1.0942	0.7055	0.084*	
C16	0.6022 (8)	1.0320 (5)	0.6543 (3)	0.0362 (14)	
C17	0.7260 (8)	1.0574 (5)	0.6166 (3)	0.0367 (14)	
C18	0.7413 (10)	1.1311 (7)	0.5647 (4)	0.060 (2)	
H18A	0.6539	1.0903	0.5200	0.090*	
H18B	0.8514	1.1469	0.5532	0.090*	
H18C	0.7286	1.2045	0.5869	0.090*	
C19	0.8315 (7)	0.9969 (5)	0.6371 (3)	0.0325 (13)	
C20	0.9860 (7)	0.9949 (5)	0.6126 (3)	0.0382 (14)	
H20A	1.0500	0.9652	0.6453	0.057*	
H20B	1.0541	1.0738	0.6135	0.057*	
H20C	0.9556	0.9445	0.5630	0.057*	
C21	0.5872 (8)	0.7215 (6)	0.5339 (3)	0.0446 (16)	
H21A	0.5519	0.7419	0.5792	0.067*	
H21B	0.5045	0.6485	0.5021	0.067*	
H21C	0.5961	0.7834	0.5093	0.067*	
C22	0.7537 (7)	0.7075 (5)	0.5509 (3)	0.0320 (13)	
C23	0.8465 (8)	0.6761 (5)	0.5009 (3)	0.0365 (14)	
C24	0.8004 (9)	0.6557 (7)	0.4202 (3)	0.057 (2)	
H24A	0.8927	0.6445	0.3994	0.086*	
H24B	0.7785	0.7231	0.4088	0.086*	
H24C	0.6998	0.5863	0.3993	0.086*	
C25	0.9900 (7)	0.6730 (5)	0.5424 (3)	0.0306 (13)	
C26	1.1374 (8)	0.6458 (6)	0.5223 (3)	0.0391 (15)	
H26A	1.2356	0.7173	0.5368	0.059*	
H26B	1.1128	0.6124	0.4696	0.059*	
H26C	1.1600	0.5900	0.5475	0.059*	
N11	0.5554 (6)	0.5762 (4)	0.8632 (3)	0.0380 (12)	
N12	0.4330 (6)	0.4886 (4)	0.8116 (3)	0.0346 (12)	
H12	0.3677	0.4994	0.7775	0.042*	
C27	0.2961 (9)	0.2742 (5)	0.7681 (4)	0.0502 (17)	
H27A	0.1842	0.2746	0.7714	0.075*	
H27B	0.3111	0.2061	0.7814	0.075*	
H27C	0.3086	0.2710	0.7182	0.075*	
C28	0.4246 (8)	0.3820 (5)	0.8189 (3)	0.0343 (13)	
C29	0.5492 (7)	0.4002 (5)	0.8791 (3)	0.0348 (14)	
C30	0.6010 (10)	0.3130 (6)	0.9091 (4)	0.058 (2)	
H30A	0.5258	0.2347	0.8815	0.088*	
H30B	0.5955	0.3252	0.9603	0.088*	
H30C	0.7157	0.3217	0.9052	0.088*	
C31	0.6268 (8)	0.5218 (5)	0.9051 (3)	0.0401 (15)	
C32	0.7669 (8)	0.5892 (6)	0.9702 (4)	0.0469 (17)	
H32A	0.8139	0.6697	0.9678	0.070*	
H32B	0.8539	0.5543	0.9709	0.070*	
H32C	0.7248	0.5880	1.0145	0.070*	

### Atomic displacement parameters (Å^2^)

**Table:**

	*U*^11^	*U*^22^	*U*^33^	*U*^12^	*U*^13^	*U*^23^
Ni1	0.0254 (4)	0.0281 (4)	0.0206 (4)	0.0071 (3)	0.0026 (3)	0.0065 (3)
S1	0.0277 (8)	0.0426 (9)	0.0462 (10)	0.0088 (7)	0.0072 (7)	0.0246 (7)
S2	0.0279 (8)	0.0463 (9)	0.0267 (8)	0.0077 (7)	−0.0017 (6)	−0.0020 (6)
N1	0.033 (3)	0.028 (2)	0.025 (3)	0.006 (2)	0.000 (2)	0.005 (2)
N2	0.032 (3)	0.032 (3)	0.021 (2)	0.005 (2)	−0.003 (2)	0.003 (2)
N3	0.029 (2)	0.029 (2)	0.018 (2)	0.006 (2)	0.0010 (19)	0.0046 (18)
N4	0.035 (3)	0.027 (2)	0.022 (2)	0.009 (2)	−0.004 (2)	0.0030 (19)
N5	0.031 (3)	0.025 (2)	0.024 (2)	0.004 (2)	0.004 (2)	0.0057 (19)
N6	0.040 (3)	0.026 (2)	0.022 (2)	0.005 (2)	0.003 (2)	0.0064 (19)
N7	0.028 (3)	0.044 (3)	0.033 (3)	0.018 (2)	0.016 (2)	0.017 (2)
N8	0.032 (3)	0.031 (3)	0.028 (3)	0.010 (2)	0.006 (2)	0.010 (2)
N9	0.021 (2)	0.039 (3)	0.029 (3)	0.013 (2)	0.007 (2)	0.014 (2)
N10	0.032 (3)	0.040 (3)	0.021 (2)	0.014 (2)	0.005 (2)	0.011 (2)
C1	0.027 (3)	0.026 (3)	0.024 (3)	0.013 (2)	0.003 (2)	0.010 (2)
C2	0.040 (3)	0.025 (3)	0.017 (3)	0.010 (2)	0.010 (2)	0.006 (2)
C3	0.036 (3)	0.031 (3)	0.030 (3)	0.007 (3)	0.001 (3)	0.001 (2)
C4	0.030 (3)	0.030 (3)	0.022 (3)	0.005 (2)	0.006 (2)	0.007 (2)
C5	0.035 (3)	0.029 (3)	0.028 (3)	0.011 (3)	0.012 (3)	0.008 (2)
C6	0.051 (4)	0.034 (3)	0.043 (4)	0.011 (3)	0.009 (3)	0.009 (3)
C7	0.038 (3)	0.034 (3)	0.022 (3)	0.014 (3)	0.007 (2)	0.008 (2)
C8	0.056 (4)	0.044 (4)	0.025 (3)	0.023 (3)	0.001 (3)	0.010 (3)
C9	0.044 (4)	0.049 (4)	0.023 (3)	0.008 (3)	0.009 (3)	0.004 (3)
C10	0.025 (3)	0.040 (3)	0.018 (3)	0.010 (2)	0.000 (2)	0.002 (2)
C11	0.031 (3)	0.034 (3)	0.022 (3)	0.013 (3)	−0.001 (2)	0.000 (2)
C12	0.040 (4)	0.036 (3)	0.034 (3)	0.008 (3)	0.003 (3)	−0.003 (3)
C13	0.032 (3)	0.030 (3)	0.029 (3)	0.008 (2)	0.003 (2)	0.007 (2)
C14	0.049 (4)	0.024 (3)	0.041 (4)	−0.001 (3)	0.007 (3)	0.004 (3)
C15	0.055 (4)	0.068 (5)	0.068 (5)	0.038 (4)	0.030 (4)	0.033 (4)
C16	0.036 (3)	0.036 (3)	0.042 (4)	0.017 (3)	0.010 (3)	0.015 (3)
C17	0.044 (4)	0.042 (3)	0.033 (3)	0.019 (3)	0.010 (3)	0.019 (3)
C18	0.070 (5)	0.067 (5)	0.061 (5)	0.030 (4)	0.027 (4)	0.038 (4)
C19	0.037 (3)	0.032 (3)	0.026 (3)	0.009 (3)	0.006 (3)	0.009 (2)
C20	0.036 (3)	0.049 (4)	0.037 (4)	0.018 (3)	0.016 (3)	0.018 (3)
C21	0.037 (3)	0.071 (5)	0.021 (3)	0.018 (3)	−0.001 (3)	0.007 (3)
C22	0.032 (3)	0.042 (3)	0.024 (3)	0.015 (3)	0.005 (2)	0.012 (2)
C23	0.043 (4)	0.045 (4)	0.023 (3)	0.017 (3)	0.008 (3)	0.008 (3)
C24	0.060 (5)	0.085 (6)	0.030 (4)	0.035 (4)	0.009 (3)	0.009 (4)
C25	0.038 (3)	0.034 (3)	0.027 (3)	0.018 (3)	0.011 (3)	0.013 (2)
C26	0.040 (4)	0.052 (4)	0.034 (3)	0.024 (3)	0.014 (3)	0.014 (3)
N11	0.039 (3)	0.037 (3)	0.032 (3)	0.005 (2)	0.006 (2)	0.011 (2)
N12	0.037 (3)	0.035 (3)	0.028 (3)	0.008 (2)	0.001 (2)	0.013 (2)
C27	0.057 (4)	0.041 (4)	0.046 (4)	0.011 (3)	0.008 (3)	0.010 (3)
C28	0.039 (3)	0.034 (3)	0.030 (3)	0.010 (3)	0.012 (3)	0.011 (3)
C29	0.035 (3)	0.040 (3)	0.037 (3)	0.012 (3)	0.014 (3)	0.022 (3)
C30	0.069 (5)	0.057 (5)	0.063 (5)	0.027 (4)	0.020 (4)	0.037 (4)
C31	0.045 (4)	0.040 (3)	0.032 (3)	0.008 (3)	0.004 (3)	0.017 (3)
C32	0.037 (4)	0.051 (4)	0.043 (4)	0.005 (3)	0.000 (3)	0.017 (3)

### Geometric parameters (Å, °)

**Table:**

Ni1—N1	2.071 (5)	C13—C14	1.495 (8)
Ni1—N2	2.065 (5)	C14—H14A	0.9800
Ni1—N3	2.128 (5)	C14—H14B	0.9800
Ni1—N6	2.111 (5)	C14—H14C	0.9800
Ni1—N8	2.120 (5)	C15—C16	1.529 (9)
Ni1—N10	2.108 (5)	C15—H15A	0.9800
S1—C1	1.632 (6)	C15—H15B	0.9800
S2—C2	1.645 (6)	C15—H15C	0.9800
N1—C2	1.171 (7)	C16—C17	1.378 (8)
N2—C1	1.177 (7)	C17—C19	1.415 (8)
N3—C4	1.347 (7)	C17—C18	1.491 (8)
N3—N4	1.354 (6)	C18—H18A	0.9800
N4—C7	1.360 (7)	C18—H18B	0.9800
N4—H4	0.8599	C18—H18C	0.9800
N5—C10	1.337 (7)	C19—C20	1.488 (8)
N5—N6	1.371 (6)	C20—H20A	0.9800
N5—H5	0.8597	C20—H20B	0.9800
N6—C13	1.358 (7)	C20—H20C	0.9800
N7—C16	1.337 (7)	C21—C22	1.498 (8)
N7—N8	1.372 (6)	C21—H21A	0.9800
N7—H7	0.8595	C21—H21B	0.9800
N8—C19	1.339 (7)	C21—H21C	0.9800
N9—C25	1.347 (7)	C22—C23	1.416 (8)
N9—N10	1.354 (6)	C23—C25	1.384 (8)
N9—H9	0.8595	C23—C24	1.496 (8)
N10—C22	1.336 (7)	C24—H24A	0.9800
C3—C4	1.489 (7)	C24—H24B	0.9800
C3—H3A	0.9800	C24—H24C	0.9800
C3—H3B	0.9800	C25—C26	1.501 (8)
C3—H3C	0.9800	C26—H26A	0.9800
C4—C5	1.411 (8)	C26—H26B	0.9800
C5—C7	1.383 (8)	C26—H26C	0.9800
C5—C6	1.509 (8)	N11—C31	1.353 (7)
C6—H6A	0.9800	N11—N12	1.355 (6)
C6—H6B	0.9800	N12—C28	1.356 (7)
C6—H6C	0.9800	N12—H12	0.8600
C7—C8	1.482 (8)	C27—C28	1.492 (8)
C8—H8A	0.9800	C27—H27A	0.9800
C8—H8B	0.9800	C27—H27B	0.9800
C8—H8C	0.9800	C27—H27C	0.9800
C9—C10	1.496 (8)	C28—C29	1.385 (8)
C9—H9A	0.9800	C29—C31	1.411 (8)
C9—H9B	0.9800	C29—C30	1.488 (8)
C9—H9C	0.9800	C30—H30A	0.9800
C10—C11	1.372 (8)	C30—H30B	0.9800
C11—C13	1.401 (8)	C30—H30C	0.9800
C11—C12	1.504 (7)	C31—C32	1.492 (8)
C12—H12A	0.9800	C32—H32A	0.9800
C12—H12B	0.9800	C32—H32B	0.9800
C12—H12C	0.9800	C32—H32C	0.9800
			
N2—Ni1—N1	175.40 (18)	C13—C14—H14A	109.5
N2—Ni1—N10	93.84 (18)	C13—C14—H14B	109.5
N1—Ni1—N10	89.50 (18)	H14A—C14—H14B	109.5
N2—Ni1—N6	88.00 (19)	C13—C14—H14C	109.5
N1—Ni1—N6	88.70 (19)	H14A—C14—H14C	109.5
N10—Ni1—N6	178.05 (18)	H14B—C14—H14C	109.5
N2—Ni1—N8	88.65 (19)	C16—C15—H15A	109.5
N1—Ni1—N8	94.61 (18)	C16—C15—H15B	109.5
N10—Ni1—N8	88.24 (18)	H15A—C15—H15B	109.5
N6—Ni1—N8	91.15 (19)	C16—C15—H15C	109.5
N2—Ni1—N3	89.78 (18)	H15A—C15—H15C	109.5
N1—Ni1—N3	87.06 (18)	H15B—C15—H15C	109.5
N10—Ni1—N3	89.98 (18)	N7—C16—C17	107.8 (5)
N6—Ni1—N3	90.68 (18)	N7—C16—C15	122.1 (6)
N8—Ni1—N3	177.55 (16)	C17—C16—C15	130.1 (6)
C2—N1—Ni1	161.9 (4)	C16—C17—C19	105.0 (5)
C1—N2—Ni1	166.9 (4)	C16—C17—C18	126.8 (6)
C4—N3—N4	104.6 (4)	C19—C17—C18	128.2 (6)
C4—N3—Ni1	136.2 (4)	C17—C18—H18A	109.5
N4—N3—Ni1	118.8 (3)	C17—C18—H18B	109.5
N3—N4—C7	113.0 (4)	H18A—C18—H18B	109.5
N3—N4—H4	123.6	C17—C18—H18C	109.5
C7—N4—H4	123.4	H18A—C18—H18C	109.5
C10—N5—N6	112.7 (4)	H18B—C18—H18C	109.5
C10—N5—H5	123.5	N8—C19—C17	110.5 (5)
N6—N5—H5	123.7	N8—C19—C20	122.0 (5)
C13—N6—N5	103.8 (4)	C17—C19—C20	127.5 (5)
C13—N6—Ni1	135.0 (4)	C19—C20—H20A	109.5
N5—N6—Ni1	120.8 (3)	C19—C20—H20B	109.5
C16—N7—N8	111.5 (5)	H20A—C20—H20B	109.5
C16—N7—H7	124.3	C19—C20—H20C	109.5
N8—N7—H7	124.2	H20A—C20—H20C	109.5
C19—N8—N7	105.2 (4)	H20B—C20—H20C	109.5
C19—N8—Ni1	135.2 (4)	C22—C21—H21A	109.5
N7—N8—Ni1	118.2 (3)	C22—C21—H21B	109.5
C25—N9—N10	113.9 (4)	H21A—C21—H21B	109.5
C25—N9—H9	122.9	C22—C21—H21C	109.5
N10—N9—H9	123.3	H21A—C21—H21C	109.5
C22—N10—N9	104.6 (4)	H21B—C21—H21C	109.5
C22—N10—Ni1	136.7 (4)	N10—C22—C23	110.4 (5)
N9—N10—Ni1	117.8 (3)	N10—C22—C21	122.2 (5)
N2—C1—S1	177.8 (5)	C23—C22—C21	127.4 (5)
N1—C2—S2	178.7 (5)	C25—C23—C22	106.0 (5)
C4—C3—H3A	109.5	C25—C23—C24	127.6 (6)
C4—C3—H3B	109.5	C22—C23—C24	126.4 (6)
H3A—C3—H3B	109.5	C23—C24—H24A	109.5
C4—C3—H3C	109.5	C23—C24—H24B	109.5
H3A—C3—H3C	109.5	H24A—C24—H24B	109.5
H3B—C3—H3C	109.5	C23—C24—H24C	109.5
N3—C4—C5	110.8 (5)	H24A—C24—H24C	109.5
N3—C4—C3	122.5 (5)	H24B—C24—H24C	109.5
C5—C4—C3	126.7 (5)	N9—C25—C23	105.2 (5)
C7—C5—C4	105.7 (5)	N9—C25—C26	122.5 (5)
C7—C5—C6	127.0 (5)	C23—C25—C26	132.3 (5)
C4—C5—C6	127.2 (5)	C25—C26—H26A	109.5
C5—C6—H6A	109.5	C25—C26—H26B	109.5
C5—C6—H6B	109.5	H26A—C26—H26B	109.5
H6A—C6—H6B	109.5	C25—C26—H26C	109.5
C5—C6—H6C	109.5	H26A—C26—H26C	109.5
H6A—C6—H6C	109.5	H26B—C26—H26C	109.5
H6B—C6—H6C	109.5	C31—N11—N12	104.3 (5)
N4—C7—C5	105.9 (5)	N11—N12—C28	113.0 (5)
N4—C7—C8	121.8 (5)	N11—N12—H12	123.6
C5—C7—C8	132.2 (5)	C28—N12—H12	123.4
C7—C8—H8A	109.5	C28—C27—H27A	109.5
C7—C8—H8B	109.5	C28—C27—H27B	109.5
H8A—C8—H8B	109.5	H27A—C27—H27B	109.5
C7—C8—H8C	109.5	C28—C27—H27C	109.5
H8A—C8—H8C	109.5	H27A—C27—H27C	109.5
H8B—C8—H8C	109.5	H27B—C27—H27C	109.5
C10—C9—H9A	109.5	N12—C28—C29	106.4 (5)
C10—C9—H9B	109.5	N12—C28—C27	121.3 (5)
H9A—C9—H9B	109.5	C29—C28—C27	132.2 (6)
C10—C9—H9C	109.5	C28—C29—C31	105.2 (5)
H9A—C9—H9C	109.5	C28—C29—C30	128.9 (6)
H9B—C9—H9C	109.5	C31—C29—C30	125.8 (6)
N5—C10—C11	107.0 (5)	C29—C30—H30A	109.5
N5—C10—C9	121.9 (5)	C29—C30—H30B	109.5
C11—C10—C9	131.1 (5)	H30A—C30—H30B	109.5
C10—C11—C13	105.9 (5)	C29—C30—H30C	109.5
C10—C11—C12	126.7 (5)	H30A—C30—H30C	109.5
C13—C11—C12	127.4 (6)	H30B—C30—H30C	109.5
C11—C12—H12A	109.5	N11—C31—C29	111.0 (5)
C11—C12—H12B	109.5	N11—C31—C32	121.1 (6)
H12A—C12—H12B	109.5	C29—C31—C32	127.8 (6)
C11—C12—H12C	109.5	C31—C32—H32A	109.5
H12A—C12—H12C	109.5	C31—C32—H32B	109.5
H12B—C12—H12C	109.5	H32A—C32—H32B	109.5
N6—C13—C11	110.6 (5)	C31—C32—H32C	109.5
N6—C13—C14	121.8 (5)	H32A—C32—H32C	109.5
C11—C13—C14	127.5 (5)	H32B—C32—H32C	109.5
			
N10—Ni1—N1—C2	−128.8 (14)	C6—C5—C7—N4	178.0 (6)
N6—Ni1—N1—C2	52.0 (14)	C4—C5—C7—C8	−175.2 (6)
N8—Ni1—N1—C2	143.1 (14)	C6—C5—C7—C8	0.8 (11)
N3—Ni1—N1—C2	−38.7 (14)	N6—N5—C10—C11	−0.2 (6)
N10—Ni1—N2—C1	160 (2)	N6—N5—C10—C9	177.4 (5)
N6—Ni1—N2—C1	−21 (2)	N5—C10—C11—C13	0.1 (6)
N8—Ni1—N2—C1	−112 (2)	C9—C10—C11—C13	−177.2 (6)
N3—Ni1—N2—C1	70 (2)	N5—C10—C11—C12	179.9 (5)
N2—Ni1—N3—C4	50.6 (5)	C9—C10—C11—C12	2.6 (10)
N1—Ni1—N3—C4	−132.7 (5)	N5—N6—C13—C11	−0.1 (6)
N10—Ni1—N3—C4	−43.2 (6)	Ni1—N6—C13—C11	172.1 (4)
N6—Ni1—N3—C4	138.6 (6)	N5—N6—C13—C14	−178.4 (5)
N2—Ni1—N3—N4	−136.9 (4)	Ni1—N6—C13—C14	−6.2 (9)
N1—Ni1—N3—N4	39.7 (4)	C10—C11—C13—N6	0.0 (7)
N10—Ni1—N3—N4	129.2 (4)	C12—C11—C13—N6	−179.8 (5)
N6—Ni1—N3—N4	−48.9 (4)	C10—C11—C13—C14	178.2 (6)
C4—N3—N4—C7	1.0 (6)	C12—C11—C13—C14	−1.7 (10)
Ni1—N3—N4—C7	−173.6 (4)	N8—N7—C16—C17	−1.0 (7)
C10—N5—N6—C13	0.2 (6)	N8—N7—C16—C15	179.5 (6)
C10—N5—N6—Ni1	−173.4 (3)	N7—C16—C17—C19	0.6 (7)
N2—Ni1—N6—C13	−129.0 (6)	C15—C16—C17—C19	−180.0 (6)
N1—Ni1—N6—C13	54.2 (6)	N7—C16—C17—C18	179.5 (6)
N8—Ni1—N6—C13	−40.4 (6)	C15—C16—C17—C18	−1.0 (12)
N3—Ni1—N6—C13	141.2 (6)	N7—N8—C19—C17	−0.6 (6)
N2—Ni1—N6—N5	42.2 (4)	Ni1—N8—C19—C17	165.1 (4)
N1—Ni1—N6—N5	−134.6 (4)	N7—N8—C19—C20	178.9 (5)
N8—Ni1—N6—N5	130.8 (4)	Ni1—N8—C19—C20	−15.4 (9)
N3—Ni1—N6—N5	−47.6 (4)	C16—C17—C19—N8	0.0 (7)
C16—N7—N8—C19	1.0 (6)	C18—C17—C19—N8	−178.9 (6)
C16—N7—N8—Ni1	−167.6 (4)	C16—C17—C19—C20	−179.5 (6)
N2—Ni1—N8—C19	−132.4 (5)	C18—C17—C19—C20	1.6 (11)
N1—Ni1—N8—C19	50.8 (6)	N9—N10—C22—C23	−1.9 (6)
N10—Ni1—N8—C19	−38.6 (5)	Ni1—N10—C22—C23	166.1 (4)
N6—Ni1—N8—C19	139.6 (5)	N9—N10—C22—C21	179.5 (5)
N2—Ni1—N8—N7	31.9 (4)	Ni1—N10—C22—C21	−12.6 (9)
N1—Ni1—N8—N7	−144.9 (4)	N10—C22—C23—C25	2.2 (7)
N10—Ni1—N8—N7	125.7 (4)	C21—C22—C23—C25	−179.2 (6)
N6—Ni1—N8—N7	−56.1 (4)	N10—C22—C23—C24	−175.4 (6)
C25—N9—N10—C22	0.9 (6)	C21—C22—C23—C24	3.2 (11)
C25—N9—N10—Ni1	−169.8 (4)	N10—N9—C25—C23	0.5 (6)
N2—Ni1—N10—C22	46.3 (6)	N10—N9—C25—C26	179.1 (5)
N1—Ni1—N10—C22	−136.8 (6)	C22—C23—C25—N9	−1.6 (6)
N8—Ni1—N10—C22	−42.2 (6)	C24—C23—C25—N9	176.0 (6)
N3—Ni1—N10—C22	136.1 (6)	C22—C23—C25—C26	−180.0 (6)
N2—Ni1—N10—N9	−146.9 (4)	C24—C23—C25—C26	−2.4 (11)
N1—Ni1—N10—N9	29.9 (4)	C31—N11—N12—C28	−0.2 (7)
N8—Ni1—N10—N9	124.6 (4)	N11—N12—C28—C29	0.0 (7)
N3—Ni1—N10—N9	−57.1 (4)	N11—N12—C28—C27	178.9 (5)
N4—N3—C4—C5	0.4 (6)	N12—C28—C29—C31	0.3 (7)
Ni1—N3—C4—C5	173.5 (4)	C27—C28—C29—C31	−178.5 (7)
N4—N3—C4—C3	−179.8 (5)	N12—C28—C29—C30	−176.4 (6)
Ni1—N3—C4—C3	−6.7 (9)	C27—C28—C29—C30	4.8 (12)
N3—C4—C5—C7	−1.5 (6)	N12—N11—C31—C29	0.4 (7)
C3—C4—C5—C7	178.7 (6)	N12—N11—C31—C32	−178.6 (6)
N3—C4—C5—C6	−177.5 (5)	C28—C29—C31—N11	−0.4 (7)
C3—C4—C5—C6	2.7 (10)	C30—C29—C31—N11	176.4 (6)
N3—N4—C7—C5	−1.9 (6)	C28—C29—C31—C32	178.5 (7)
N3—N4—C7—C8	175.6 (5)	C30—C29—C31—C32	−4.7 (11)
C4—C5—C7—N4	2.0 (6)		

### Hydrogen-bond geometry (Å, °)

**Table:**

*D*—H···*A*	*D*—H	H···*A*	*D*···*A*	*D*—H···*A*
N5—H5···N11	0.86	2.15	2.970 (8)	159
N7—H7···S2^i^	0.86	2.66	3.441 (6)	152
N9—H9···S1^ii^	0.86	2.59	3.348 (6)	148
N12—H12···S1	0.86	2.49	3.292 (7)	156
C3—H3A···N2	0.98	2.57	3.338 (9)	135
C14—H14A···N1	0.98	2.50	3.324 (9)	141
C20—H20A···N1	0.98	2.49	3.371 (8)	150
C21—H21A···N2	0.98	2.48	3.326 (8)	145

Symmetry codes: (i) *x*−1, *y*, *z*; (ii) *x*+1, *y*, *z*.
